# Femtosecond Laser-Processing of Pre-Anodized Ti-Based Bone Implants for Cell-Repellent Functionalization

**DOI:** 10.3390/nano11051342

**Published:** 2021-05-20

**Authors:** Martina Muck, Benedikt Wolfsjäger, Karoline Seibert, Christian Maier, Shaukat Ali Lone, Achim Walter Hassel, Werner Baumgartner, Johannes Heitz

**Affiliations:** 1 Institute of Applied Physics, Johannes Kepler University Linz, Altenberger Strasse 69, 4040 Linz, Austria; martina.muck@jku.at (M.M.); benedikt.wolfsjaeger@gmail.com (B.W.); 2 Hofer GmbH & Co KG, Jahnstrasse 10-12, 8280 Fürstenfeld, Austria; karoline.seibert@hofer-medical.com (K.S.); christian.maier@hofer-medical.com (C.M.); 3 Institute of Chemical Technology of Inorganic Materials, Johannes Kepler University Linz, Altenberger Strasse 69, 4040 Linz, Austria; shaukat_ali.lone@jku.at (S.A.L.); achimwalter.hassel@jku.at (A.W.H.); 4 Institute of Biomedical Mechatronics, Johannes Kepler University Linz, Altenberger Strasse 69, 4040 Linz, Austria; werner.baumgartner@jku.at

**Keywords:** ultrafast laser-processing, laser-induced microstructures and nanostructures, cell activation, cell-repellent surfaces, medical implants

## Abstract

Microstructures and nanostructures can be used to reduce the adhesion of the cells on the auxiliary material. Therefore, the aim of our work was to fabricate laser-induced hierarchical microstructures and nanostructures by femtosecond laser-treatment (wavelength 1040 nm, pulse length 350 fs, repetition rates in the kHz range) to reduce the cell adhesion. Additionally, surface chemistry modification by optimized electrochemical anodization was used to further reduce the cell adhesion. For testing, flat plates and bone screws made of Ti-6Al-4V were used. Bone-forming cells (human osteoblasts from the cell line SAOS-2) were grown on the bone implants and additional test samples for two to three weeks. After the growth period, the cells were characterized by scanning electron microscopy (SEM). While earlier experiments with fibroblasts had shown that femtosecond laser-processing followed by electrochemical anodization had a significant impact on cell adhesion reduction, for osteoblasts the same conditions resulted in an activation of the cells with increased production of extracellular matrix material. Significant reduction of cell adhesion for osteoblasts was only obtained at pre-anodized surfaces. It could be demonstrated that this functionalization by means of femtosecond laser-processing can result in bone screws that hinder the adhesion of osteoblasts.

## 1. Introduction

In medicine, osteosynthesis is the stabilization of broken bones or fragments of bones by, e.g., implanting Ti-based bone plates or screws via an invasive operation. However, the implant material needs to be removed (in most cases) after several months when the bone is nearly or fully healed. The material can be overgrown with bone-forming cells (so called osteoblasts) during this time and be completely integrated into the mineralized extracellular matrix produced by these cells. The removal can then be accompanied by destruction of the newly grown bone matrix.

Controlled cell-adhesion on titanium (Ti) surfaces due to femtosecond-laser induced microstructures and nanostructures has been investigated by several groups in the last few years [1,2,3]. This interest is triggered by the fact that Ti and Ti-alloys are often used materials for medical implants, for instance in dentistry, orthopedics, and for cardio-vascular applications. The main focus up to now was improved osseo-integration (e.g., the integration into the jaw bone for dental implants) due to the activation of bone-forming osteoblasts for enhanced production of bone material or the differentiation of mesenchymal stem cells into osteoblast cells, which can be induced by certain nano-features [4,5]. However, for many other applications, the medical implants may have to be removed from the patient’s body after several months or years. In this case, a complete ingrowth into the tissue should be avoided. One example is the Ti-based medical bone plates or screws, as described above.

Therefore, ideas for preventing a full ingrowth of these implants would be of large interest. It has been found [6,7] that femtosecond laser-induced conical micrometer spikes and surface periodical nanometer ripples on silicon can be used to tune cell adhesion, though with distinct differences between the different types of adherent cells. It is assumed that the cells are not able to sufficiently adjust their shape to the micrometer surface structure, and therefore a limitation of the contact area between the cell and substrate is achieved. Furthermore, the presence of nanometer sized periodic structures at the surface supposedly limits the number of focal adhesions on the tips of the spikes. Here, the exact geometry of the combined microstructures and nanostructures seems to be of great relevance, as adherent cells can grow well on certain nanostructured materials, which can even be used as functional substrates [8,9]. Another important feature for the cell-substrate interaction is the oxide layer on top of the Ti or Ti alloy, which can be thickened in a controlled manner by electrochemical anodization [10]. This treatment is applied to many Ti-based medical implants due to the chemical inertness, corrosion resistance, and mechanical stability of the resulting layers and also due to the colorful appearance of these layers, which are often employed for marking and decoration purposes [11]. Additionally, electrochemical approaches for controlling the adsorption of certain extracellular matrix proteins, such as collagen, on a Ti substrate have been found [12]. Our recent articles [13,14] revealed that femtosecond laser-processing and sub-sequent electrochemical anodization of flat Ti surfaces can lead to a reduction of cell-substrate adhesion forces, at least for fibroblast cells, which are typical for connective and scar tissue.

In this work, we extend the focus of our studies to the repellence of bone-forming osteoblast cells. We combine and optimize femtosecond laser-structuring and electrochemical processing with respect to their capability of reducing the adhesion of these cells on a cylindric Ti-based sample with screw windings. By these treatments, the cell-surface contact is reduced in order to allow a safe explantation of the screws, after complete healing of the fracture. Our work hypothesis was that cell-repellence for bone-forming osteoblasts can be obtained by a similar treatment to that used for fibroblasts in our previous work [13,14].

## 2. Materials and Methods

As samples, we used either flat ASTM F136 Ti-6Al-4V samples provided by the company Zapp Precision Metals GmbH (Schwerte, Germany) with an area of 20 × 20 mm^2^ and a thickness of 1 mm or pre-anodized ASTM F136 Ti-6Al-4V bone screws of the commercial product line of Hofer GmbH & Co KG (Fürstenfeld, Austria) with a length of 17 mm and a diameter of 2.5 mm. The bone screws had an intensive blue or golden color due to the anodization in an acid electrolyte (based on phosphoric acid). In addition to the laser-treatment, some of the flat ASTM F136 Ti-6Al-4V samples were anodized in 0.1 M sulfuric acid at an anodization voltage of up to 10 V, which resulted in a brown color of the samples. Anodization is an established electrochemical process by means of which a relatively thick oxide layer is grown on valve metal surfaces, like Ti-based materials, which have a native oxide layer of a few nm in any case. As is shown in the Figure S1 in the supplement material, the thickness of the layer, reaching from several 10 to 100 nm, can be estimated by measuring the anodization current, depending on the applied voltage E_SHE_. This oxidation increases the resistance against corrosion and can result in better mechanical properties and improved biocompatibility and is therefore used for many Ti-based medical implants. This thick oxide layer is also responsible for the intensive coloration of the anodized surfaces (see Figure S2 in the Supplementary Materials).

**Figure 1:**
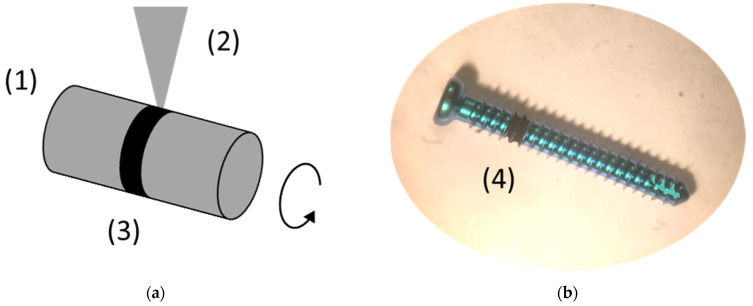
(**a**) Schematics of the laser-structuring of a cylindrical sample. The sample (1) was rotated around its the symmetry axis during the surface-processing with a focused femtosecond laser beam. The laser beam is shown schematically as a triangle (2), the processed surface in black (3). (**b**) Bone screw with a dark femtosecond laser-processed ring (4).

**Figure 2:**
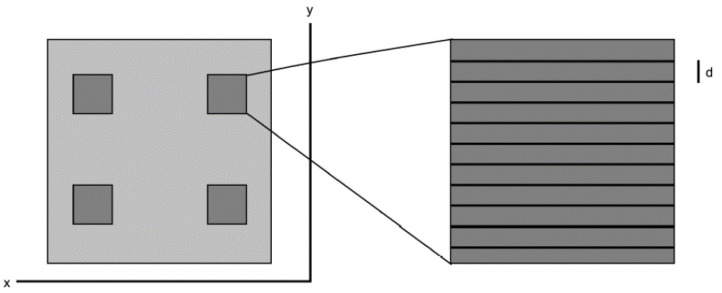
Schematics of the laser-structuring of a flat sample. The sample has four laser-processed areas (shown as darker squares). Each processed area was scanned line-by-line, resulting in a meander-pattern with a line distance d.

The laser-treatment was performed with a Yb-based amplified femtosecond laser-system (Spirit 1040-16 HE, Spectra Physics, Darmstadt, Germany) with a wavelength λ = 1040 nm, pulse length τ = 350 fs, a typical pulse energy E up to 110 µJ, and a repetition rate of up to 200 kHz. Two linear positioning stages were used to obtain a lateral movement of the samples during the irradiation. Cylindrical samples, i.e., the bone screws, were rotated with a third stepping- or linear-motor around their axis of symmetry (Figure 1). Flat samples were scanned line-by-line (Figure 2). The laser beam was focused onto the sample by a lens with a focal length of F = 200 mm, resulting in a Gaussian-shaped beam-profile with a diameter of about 100 μm, FWHM (full width of half maximum). The beam-profile was evaluated using a digital camera. The irradiation parameters (i.e., the velocity of the laser beam at the sample surface, the line distance, and the mean energy density) were adjusted to obtain a regularly ordered surface structure consisting of about 10 to 15 μm high cone-like microstructures, covered with a regular parallel ripple pattern with a periodicity of several hundred nm (also known as laser-induced periodic surface structures or LIPSS).

For the cell tests, we used bone-forming cells (osteoblasts) of the commercially available human cell line SAOS-2 (provider DSMZ—Deutsche Sammlung von Mikroorganismen und Zellkulturen GmbH, Braunschweig, Germany). The cells were cultivated in an established cell culture medium in an incubator with a water vapor saturated atmosphere with 5% CO_2_ content at 37 °C and were divided at a ratio of 1:10, once a week. The experiments with oxidized and laser-treated samples as well as with control samples were performed with typical culture times of 2 to 3 weeks. After disinfection with ethanol, a set of samples were placed in a Petri dish with cells and culture medium, thus the samples were completely covered with liquid. After the chosen cultivation time in the incubator, the cells at the samples were fixated and dehydrated. In detail, the cells were initially fixed overnight with 6% glutardialdehyde (GA; Merck, Darmstadt, Germany) in phosphate-buffered saline (PBS) and sub-sequently dehydrated with the help of ascending ethanol series (30%, 40%, 50%, 70%, 80%, 90%, 96%, 3 × 100%) for 30 min each. The samples were repeatedly transferred 3 times into 100% hexamethyl-disilazane (HMDS; Merck). After the overnight evaporation of HMDS, the samples were sputter-coated with gold and the cell density was evaluated by means of scanning electron microscopy (SEM).

## 3. Results

In our previous work [13,14], the cell tests were always performed with fibroblasts. For this publication, we used osteoblasts. As the effect of laser-induced microstructures and nanostructures in combination with electrochemical anodization on the adhesion of osteoblasts was unknown, we tested Ti-6Al-4V samples with different preparation parameters, including those which resulted before in nearly complete cell-repellence of fibroblasts.

**Figure 3:**
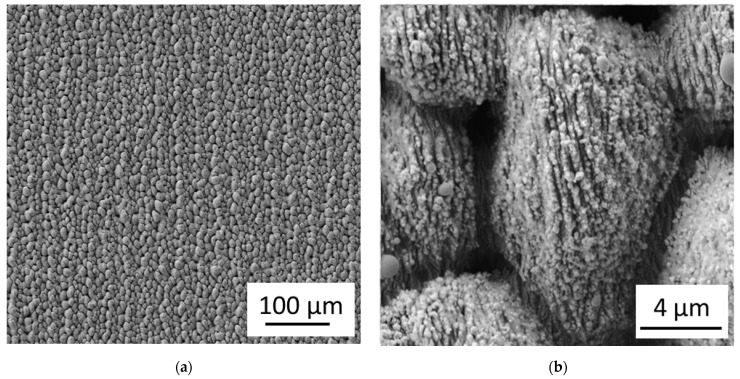
Femtosecond laser-induced microstructures and nanostructures: (**a**) SEM image of laser-induced microstructures on a flat Ti-6Al-4V sample; (**b**) Magnification of (**a**) with laser-induced nanostructures.

Figure 3 shows the femtosecond laser-induced microstructures and nanostructures obtained with the Spirit 1040-16 HE laser. The laser pulse energy was E = 70 µJ, the velocity of the positioning stage was v = 350 µm/s, the repetition rate was f = 1 kHz, and the line distance was d = 30 µm, resulting in a fluence of ϕ ≈ 1.65 J/cm^2^ and an effective number of laser pulses per area of N_eff_ ≈ 300. The laser beam was linearly polarized with the polarization direction perpendicular to the direction of the ripples.

**Figure 4:**
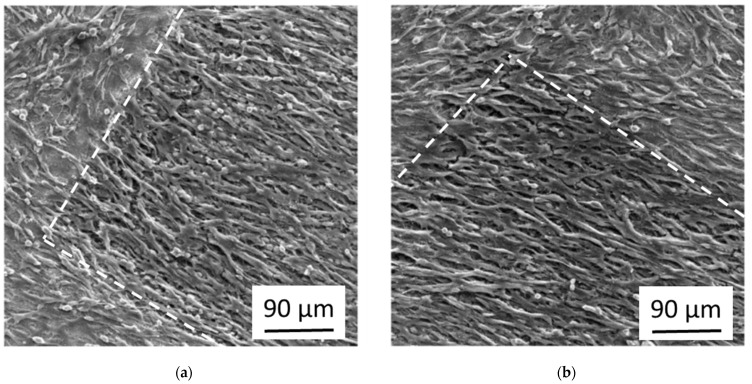
SEM images of the growth of osteoblasts at femtosecond laser-processed Ti-6Al-4V samples with and without sub-sequent anodization. The boundary of the laser-irradiated areas at the samples is indicated by a white dashed line. (**a**) Femtosecond laser-processing without anodization; (**b**) Femtosecond laser-processing followed by anodization.

Figure 4 shows the growth of osteoblasts after 20 days in culture on flat Ti-6Al-4V samples laser-processed with the parameters mentioned above (with and without sub-sequent anodization in 0.1 M sulfuric acid at a voltage of E_SHE_ = 10 V). The white line shows the boundary between the laser-processed area and the flat untreated sample surface. The laser-processed area is top/right for the image in Figure 4a and bottom in Figure 4b. For both samples, the osteoblasts at the not laser-processed areas grow in dense confluent multilayers, while the osteoblasts at the laser-processed areas still cover the surface densely, but are oriented parallel to the (non-visible) nano-ripples below them. The additional anodization had no obvious effect on the cell growth.

**Figure 5:**
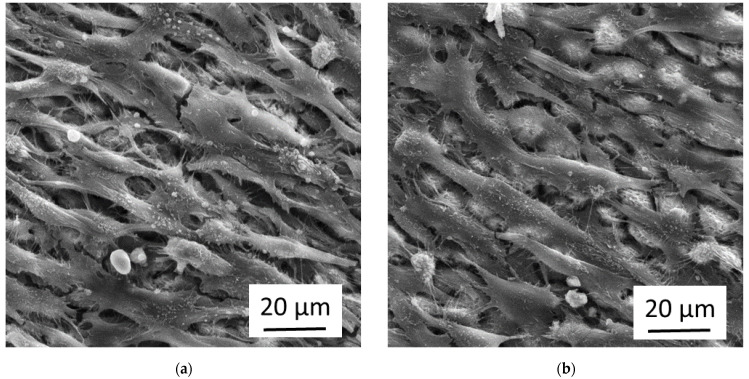
SEM images with higher magnification of the growth of osteoblasts at femtosecond laser-processed Ti-6Al-4V samples with and without sub-sequent anodization. (**a**) Femtosecond laser-processing without anodization; (**b**) Femtosecond laser-processing followed by anodization.

Figure 5 shows the osteoblasts of Figure 4 in higher magnification. It can be seen that the cells grow in multilayers and are elongated along the (not visible) direction of the nano-ripples. In some places, one sees the (bright) laser-induced nanostructures and microstructures below the cells. The cells form some cell-protrusions (i.e., filopodia). A close look shows that additionally to the cells, the cell protrusions, and the laser-induced structures, there is a considerable amount of fibrous material.

Several laser-processed rings were produced at pre-anodized bone screws in a set-up, which is schematically shown in Figure 1. Following laser irradiation parameters were used: laser pulse energy E = 70 µJ or 110 µJ, velocity of the positioning stage v = 350 µm/s, repetition rate f = 1 kHz. The laser-processing resulted in quasi-periodic microstructures covered with nano-ripples, especially on the areas between the screw windings which were oriented normal to the laser beam direction. Less pronounced structures were formed at the flanks of the screw windings.

**Figure 6:**
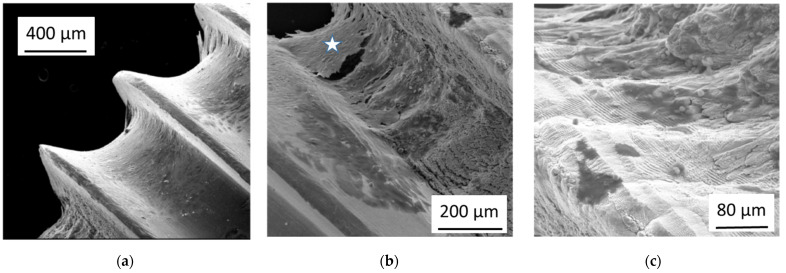
Electron microscope images of (**a**) a pre-anodized Ti-based bone screw overgrown by osteoblasts after 3 weeks in culture and (**b**) of a femtosecond laser-treated area on the same screw, where many areas remain cell-free. (**c**) shows a detail of (**b**) in higher magnification.

The effect of the laser-treatment of pre-anodized bone screws on the osteoblast adhesion is shown in Figure 6. For the pre-anodized bone screw without laser-treatment, as shown in Figure 6a, the surface is covered with a dense confluent layer of osteoblasts, which start to grow into the third dimension at the rim of the screw windings. Many areas of the laser-treated areas on the same screw (Figure 6b) remain cell-free. Instead, the hierarchical laser-induced microstructures and nanostructures become visible. Some of the cells (marked by a white star) grow together and largely lose the contact to the surface. Figure 6c shows a higher magnification of the laser-treated area. Only a few isolated cells (darker) remain on the laser-treated surface (brighter), showing a nano-ripple pattern which is characteristic for femtosecond laser-processing using linearly polarized light. There was no obvious difference of the cell growth between rings with the two different laser pulse energies.

## 4. Discussion

The fibrous material in Figure 5 is probably collagen, which is in mineralized form the main constituent of the bone matrix that is physiologically built up by the osteoblasts for instance during the healing process of broken bones. We performed immunostaining tests for collagen Type I and indeed found a strong increase of the collagen signal of osteoblasts grown on femtosecond laser-processed and subsequently anodized Ti-6Al-4V samples compared to cells grown on flat only anodized surfaces of the same material (see Figure S3 in the Supplementary Materials).

The conditions used in the Figure 4b and Figure 5b (i.e., femtosecond laser-processing combined with sub-sequent anodization) resulted in our previous work with fibroblasts [13,14] in nearly complete cell-repellence, while the osteoblasts still grow at these surfaces, though with a clearly distinct morphology than the cells on flat not laser-processed areas nearby. They also show a high activation level indicated by the formation of cell protrusions and the generation of extracellular matrix material. Both cell types, osteoblasts and fibroblasts, are closely related with mesenchymal origin. But it is known that they react differently to topographical stimuli of surface structures in the micrometer and nanometer range and that the differentiation of mesenchymal stem cells can be driven into the osteoblast direction by these stimuli, while flat surfaces promote the differentiation into the fibroblast direction [4,5,15]. This could be an important part of the explanation of why the same conditions repel fibroblasts but activate osteoblasts.

In our previous work, we showed that the same conditions that led to cell-repellence of fibroblasts at laser-processed and sub-sequently anodized Ti-6Al-4V [11] had the similar effect on pure Ti samples [14]. Also, for the osteoblasts, we performed experiments on femtosecond laser-processed pure Ti samples and found the same results as with Ti-6Al-4V. Nearly complete cell-repellence of osteoblasts could only be obtained for pre-anodized and then femtosecond laser-processed Ti samples while femtosecond laser-processing with or without sub-sequent anodization led to cell-coverage and activation of the osteoblasts.

Both treatments, anodization and femtosecond laser-processing, lead to a considerable increase of the native oxide layer on Ti-based materials, resulting in layers with a thickness in the order of 100 nm [10,11,16]. However, the characteristic of both oxides is different. The anodization leads to much denser oxides with less defects, while the oxide layers induced by the femtosecond laser-processing in air results in more porous not perfect layers. For this reason, it is possible to further oxidize laser-processed areas by anodization, as described by Lone et al. in [10]. On the other hand, the femtosecond laser-processing results in material removal and structural and topographical reorganization of the surface on a 10 to 15 µm scale. Thus, the surfaces which are first anodized and then femtosecond laser-processed should result in combined microstructures and nanostructures with a less dense and, on the nanometer scale, inhomogeneous oxide layer, while a reversal of the process steps leads to a similar surface structure but with a dense oxide layer. The inhomogeneities of the oxide on the nanoscale may be the reason for the decreased adhesion of the osteoblasts, which may react to this kind of stimulus. However, there must be also an influence of the anodization which is likely due a reduction of the oxide defects, i.e., a repair of the thermally stressed oxide film, as without it, we obtained no cell-repellence effect for the osteoblasts.

We found clear differences in the behavior of bone-forming osteoblasts and fibroblasts on the surfaces with combined femtosecond laser-processing followed by electrochemical anodization. This effect was surprising for us and rebutted our work hypothesis from the Section 1. The reason for this is probably that osteoblasts react to certain surface stimuli by an activation and increased collagen production (and not by decreased attachment as the fibroblasts) due to their physiologic role. They are optimized to grow three-dimensionally in a hard microstructured and nanostructured environment, i.e., the spongeous matrix of the trabecular bone, and react to external and internal stimuli, like stress fields, by bone-formation. Fibroblasts have other physiological roles, e.g., to form scar tissue or encapsulations, and are adapted to grow two-dimensionally in a smooth and soft surrounding. However, by using samples which were pre-anodized before femtosecond laser-treatment, we were able to find a second parameter window, which resulted in repellence of osteoblasts as well.

## 5. Conclusions

The Ti-alloy Ti-6Al-4V is often used for medical bone implants. We showed that femtosecond laser irradiation of this material can result in the formation of conical microstructures, which are covered by sub-wavelength laser-induced periodic surface ripples. We could demonstrate for the first time that these laser-induced microstructures and nanostructures at pre-anodized bone screws made of this material form a surface that is repellent for bone-forming osteoblast cells. These surfaces promise applications in the field of medical bone implants for fixation of broken bones, which have to be removed from the body some months after the implantation and shall not be fully integrated into the new-grown bone matrix. A reversal of the processing steps, i.e., femtosecond laser-processing followed by anodization, can result in surfaces with laser-induced structures which activate the osteoblast for increased generation of extracellular matrix material, which may be beneficial for implants that have to remain permanently in the body.

## 6. Patents

There is a pending patent application on results reported in this manuscript.

## Data Availability

No new data were created or analyzed in this study. Data sharing is not applicable to this article.

## Supplementary Materials

The following are available online at https://www.mdpi.com/article/10.3390/nano11051342/s1, Figure S1: Cyclic Voltammograms; Figure S2: Pre-anodized Bone Implants; Figure S3: Collagen Type I Immunostaining.
