# Supplementary material for: Femtosecond Laser-Processing of Pre-Anodized Ti-Based Bone Implants for Cell-Repellent Functionalization
Source: Nanomaterials (Basel). 2021 May 20;11(5):1342. doi: 10.3390/nano11051342 (PMC8160909; doi:10.3390/nano11051342)
Supplement: Supplementary file 1 [file nanomaterials-11-01342-s001.zip › nanomaterials-1217948-supplementary.pdf]

nanomaterials

Supplementary Materials of

Femtosecond Laser-Processing of Pre-Anodized Ti-Based Bone Implants for Cell-Repellent Functionalization

Martina Muck [1], Benedikt Wolfsjäger [1], Karoline Seibert [2], Christian Maier [2], Shaukat Ali Lone [3], Achim Walter Hassel [3], Werner Baumgartner [4] and Johannes Heitz [1,*]

1 Institute of Applied Physics, Johannes Kepler University Linz, Altenberger Strasse 69, 4040 Linz, Austria; martina.muck@jku.at (M.M.); benedikt.wolfsjaeger@gmail.com (B.W.)
2 Hofer GmbH & Co KG, Jahnstrasse 10-12, 8280 Fürstenfeld, Austria; karoline.seibert@hofer-medical.com (K.S.); christian.maier@hofer-medical.com (C.M.)
3 Institute of Chemical Technology of Inorganic Materials, Johannes Kepler University Linz, Altenberger Strasse 69, 4040 Linz, Austria; shaukat_ali.lone@jku.at (S.A.L.); achimwalter.hassel@jku.at (A.W.H.)
4 Institute of Biomedical Mechatronics, Johannes Kepler University Linz, Altenberger Strasse 69, 4040 Linz, Austria; werner.baumgartner@jku.at
* Correspondence: johannes.heitz@jku.at; Tel.: +43-732-2468-9404

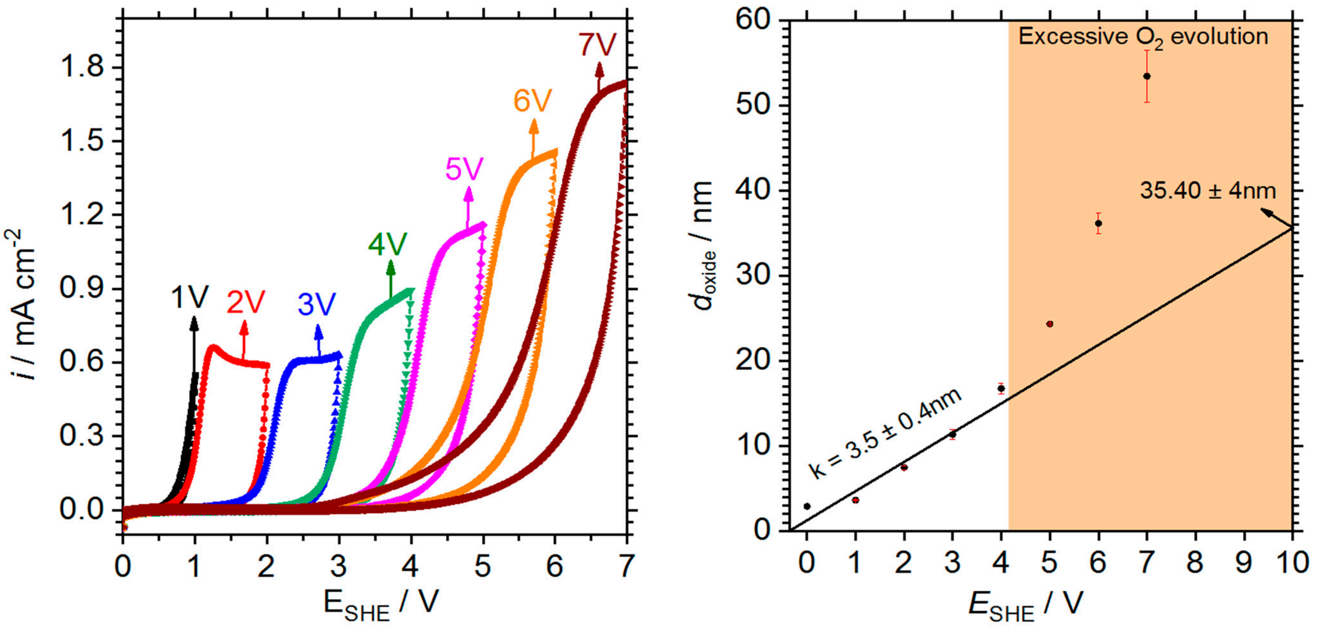

(**a**) (**b**)

Figure S1. Cyclic Voltammograms obtained by 1 V step increment in the voltage up to a maximum of (**a**) 7 V for mirror polished Ti-6Al-4V alloy surface. (**b**) Thickness estimation of anodized oxide layer formed on polished Ti-6Al-4V alloy surface after each increment in the voltage. The oxidation of the surface starts at a voltage E_{SHE} of about 1.2 V, which is accompanied by excessive O_2 evolution for voltages of about E_{SHE} = 4.3 V and higher. This figure adapted from [1] (see below).

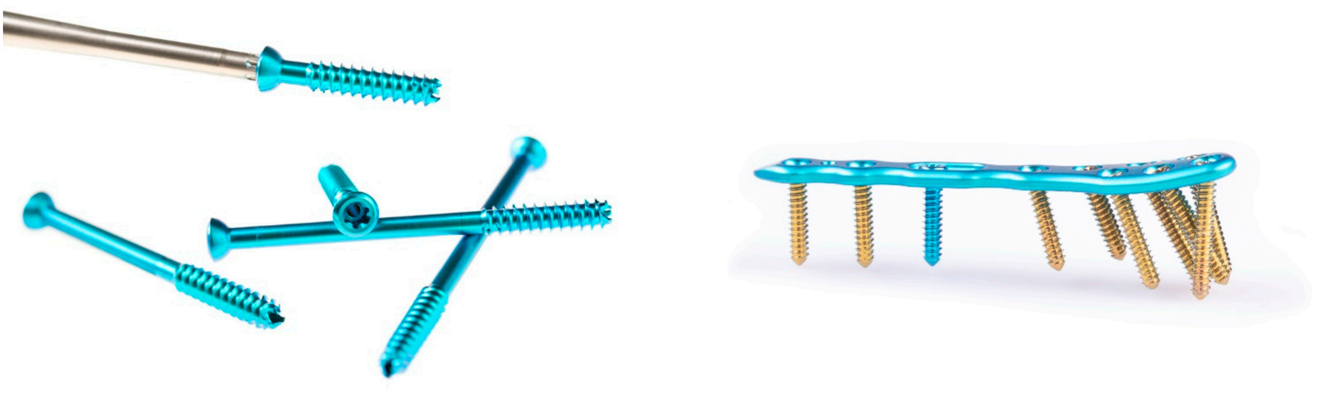

(**a**) (**b**)

Figure S2. Pre-anodized Bone Implants**:** Commercially available Ti-based bone screws and bone plates from the product line of Hofer GmbH & Co KG (Fürstenfeld, Austria, https://www.hofer-medical.com/). (**a**) Screws that show an intensive blue color due to pre-anodization, as most of the screws used in our experiments; (**b**) Blue and gold colored screws, screwed into a bone plate, that were pre-anodized under two different conditions.

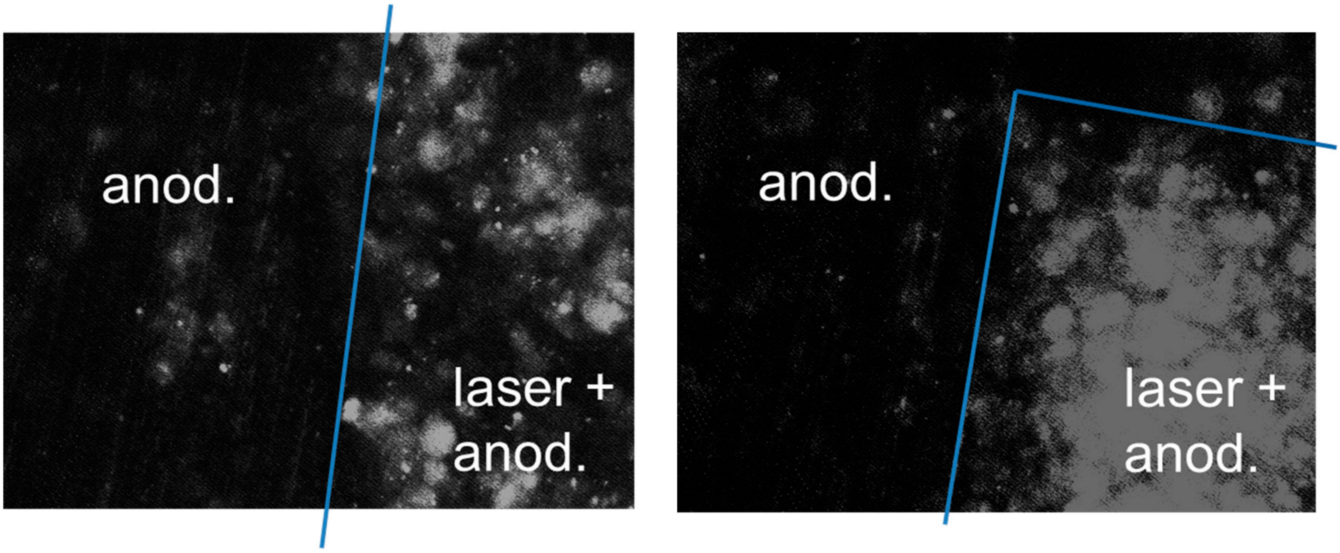

(**a**) (**b**)

Figure S3. Collagen Type I Immunostaining: Fluorescence microscope images of osteoblasts on laser-structured and/or anodized Ti-6Al-4V alloy samples stained against collagen Type I. Bright color corresponds to high fluorescence. The blue lines are the border between areas which were femtosecond laser-processed and sub-sequently anodized (laser + anod.) and areas which were anodized (anod.), respectively. Figures (**a**) and (**b**) show the results from two different samples.

References

1. Lone, S.A.; Muck, M.; Fosodeder, P.; Mardare, C.C.; Florian, C.; Weth, A., Krüger, J.; Steinwender, C.; Baumgartner, W.; Bonse, J., Heitz, J.; Hassel, A.W.; *Phys. Stat. Solidi A* **2020**, *217*, 1900838. doi:10.1002/pssa.201900838
